# Absent gallbladder discovered during evaluation for biliary pain: case report and literature review

**DOI:** 10.1186/s12893-026-03996-9

**Published:** 2026-07-06

**Authors:** Hocine Hamdi, Soumaya Belhoul, Sjaak Pouwels

**Affiliations:** 1 Department of Surgery, Public Hospital Establishment of Beni Aziz, Setif, Algeria; 2 Department of General Surgery, Saâdna Mohamed Abdennour University Hospital, University of Ferhat Abbes, Setif, Algeria; 3 Department of Surgery, Bielefeld University, Medical School and University Medical Center OWL, Campus Hospital Lippe, Röntgenstraße 18, Detmold, NRW 32756 Germany; 4 Department of Surgical Oncology, Transplant Surgery and General Surgery, Medical University of Gdansk, Gdansk, Poland

**Keywords:** Gallbladder agenesis, Biliary colic, Diagnostic laparoscopy, MRCP, Biliary tract malformations, Case report

## Abstract

**Introduction:**

Gallbladder Agenesis (GA) is a rare congenital anomaly of the biliary tract with reported incidence estimates at 0.03% in general clinical series. Although many patients are asymptomatic, up to half may present with biliary-type symptoms, creating a diagnostic challenge; routine imaging such as ultrasound or Hepatobiliary Iminodiacetic Acid scan (HIDA) may be misleading, and Magnetic Resonance Cholangio-Pancreatography (MRCP) is often required to establish the diagnosis and avoid unnecessary surgery.

**Case presentation:**

We report the case of an adult patient presenting with mild biliary-type pain clinically suggestive of gallstone disease. Initial abdominal ultrasonography was inconclusive, failing to clearly visualize the gallbladder and raising suspicion of a contracted or scleroatrophic gallbladder. Further evaluation with Magnetic Resonance Cholangiopancreatography (MRCP) demonstrated complete absence of the gallbladder with compensatory dilation of the common bile duct (CBD), confirming the diagnosis of GA.

The patient was managed conservatively with symptomatic treatment and dietary modifications, leading to significant clinical improvement and a significant regression of symptoms.

**Conclusion:**

GA is an uncommon but important differential diagnosis in patients presenting with biliary-type pain when the gallbladder is not visualized on ultrasonography. In cases of diagnostic uncertainty, early use of MRCP is crucial to accurately define biliary anatomy and may prevent unnecessary surgical exploration and associated iatrogenic biliary injury. A structured diagnostic approach based on early cross-sectional imaging is essential to reduce diagnostic delay and avoid potentially preventable operative complications.

This case report has been reported in line with the SCARE Criteria [1].

## Introduction

GA is a rare congenital anomaly of the biliary system characterized by the absence of the gallbladder and, frequently, the cystic duct. The reported incidence in the general population ranges from approximately 0.01% to 0.06% of individuals in large clinical series and case reports [2].

Although many individuals with GA remain asymptomatic, a substantial proportion -up to 50%- may present with symptoms mimicking biliary colic or chronic cholecystitis, such as right upper quadrant abdominal pain, nausea, or dyspepsia [3].

The diagnosis of GA is challenging because routine imaging modalities such as ultrasonography are frequently inconclusive or misleading, often suggesting a contracted gallbladder or cystic duct obstruction [4]. This has been reported in multiple case series and reviews, where initial ultrasound findings were interpreted as contracted or fibrotic gallbladder and advanced tests such as HIDA scans or Endoscopic Retrograde Cholangiopancreatography (ERCP) and HIDA may falsely suggest cystic duct obstruction rather than agenesis, with many cases ultimately diagnosed intra-operatively or on MRCP [5].

As a result, the anomaly is often identified intraoperatively or incidentally on advanced imaging such as MRCP, which offers superior delineation of the biliary anatomy and may prevent unnecessary surgical exploration [6].

Given the clinical overlap with more common biliary conditions and the potential for misdiagnosis, heightened awareness and structured diagnostic evaluation, including the selective use of MRCP, are essential to guide appropriate patient management and avoid risks associated with unwarranted surgical procedures [3, 6].

## Case presentation

A 56-year-old female with no significant past medical, surgical or allergies history and no relevant family history. The patient reported no history of medication use, alcohol consumption, or smoking. presented with recurrent right upper quadrant abdominal pain radiating to the right shoulder as the Chief Complaint. The pain was predominantly postprandial and progressively increasing in frequency. There were no associated symptoms including fever, jaundice, nausea, vomiting, or weight loss.

Physical examination was unremarkable for complicated gallbladder disease. The abdomen was soft, with mild right upper quadrant tenderness only, and no guarding, rebound tenderness, or palpable mass. Initial laboratory investigations revealed mild normochromic normocytic anemia and a mild isolated elevation of gamma-glutamyl transferase (GGT), while pancreatic enzymes and inflammatory markers were within normal limits (Table 1).

**Table 1:** Laboratory values, normochromic normocytic anemia, and upper limit GGT elevation are noted

Parameter (hematology)	Result	Reference range
Hemoglobin (g/dL)	11.2	12–16 (female), 13–17 (male)
MCH (pg)	31	27–33
MCV (fL)	81	80–100
WBC (×10³/µL)	5.6	4.0–10.0
Eosinophils (%)	1.2	1–6
Platelets (×10³/µL)	258	150–450

**Parameter (biochemistry)**	**Result**	**Reference range**
Blood glucose (g/L)	0.90	0.70–1.10
Blood glucose (mg/dL)	91	70–110
Serum creatinine (mg/dL)	0.7	0.6–1.2
Blood urea (g/L)	0.31	0.15–0.45
Sodium (mEq/L)	137	135–145
Potassium (mEq/L)	3.9	3.5–5.0
Prothrombin activity (%)	89	70–100

**Parameter (Liver function tests)**	**Result**	**Reference range**
ALT (U/L)	13.3	0–40
AST (U/L)	20.6	0–40
Total bilirubin (mg/dL)	0.8	0.2–1.2
GGT (U/L)	37	5–36
ALP (U/L)	110	40–130

**Fig. 1:**
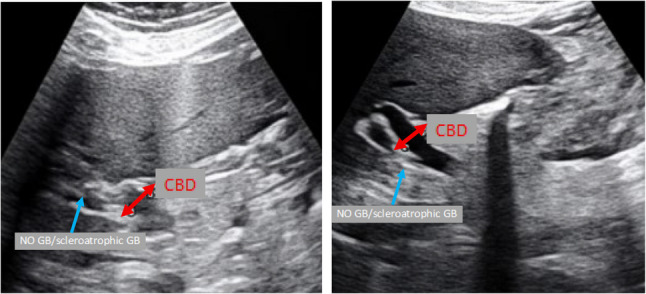
Abdominal ultrasonography demonstrating an absent/hypotrophic gallbladder appearance (blue arrow) associated with CBD dilation (red arrow), measuring 11.4 mm

**Fig. 2:**
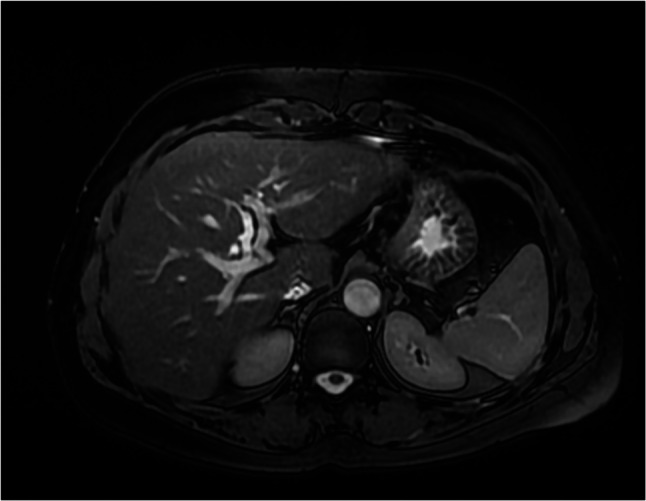
MRCP Axial T2-weighted MRCP image demonstrating hyperintense bile within the intrahepatic biliary ducts and intermediate-to-hypointense liver parenchyma relative to bile, with no identifiable fluid-filled gallbladder structure

**Fig. 3:**
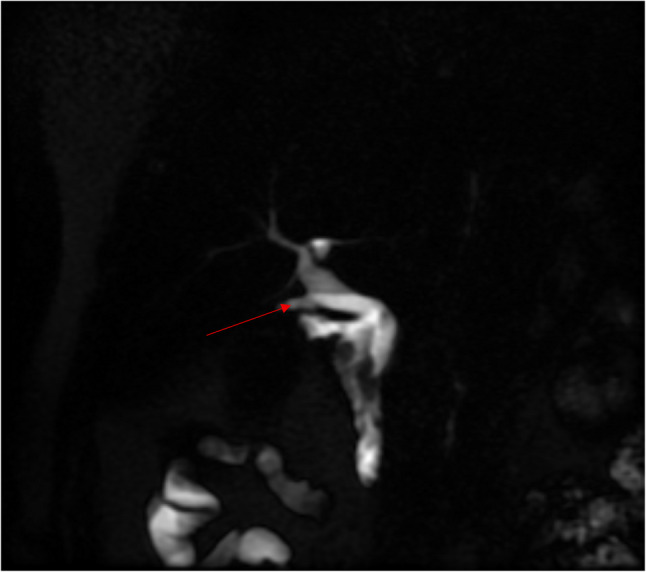
Coronal T2-weighted MRCP image demonstrating the biliary tree as hyperintense fluid-filled structures. No gallbladder lumen is visualized. A short blind-ending cystic duct remnant is identified (arrow)

**Fig. 4:**
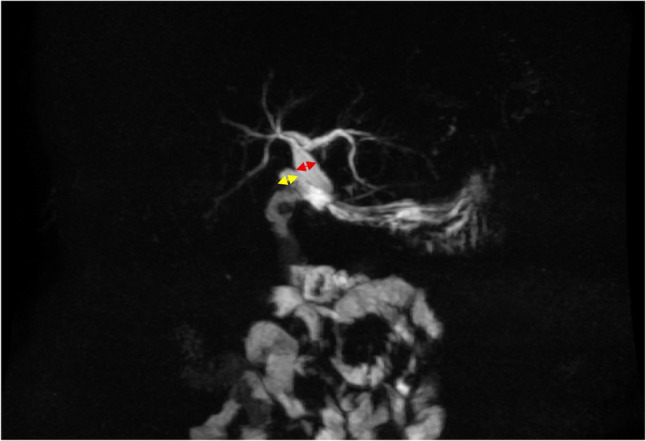
Coronal T2-weighted MRCP image demonstrating dilatation of the CBD (red measurement arrow: 10.2 mm), without visualization of the gallbladder. A short cystic duct remnant is again identified (yellow arrow: 7 mm)

Abdominal ultrasonography demonstrated inconclusive findings, described as a scleroatrophic gallbladder, with biliary ductal dilation measuring 11.4 mm and no evidence of cholelithiasis (Fig. 1). Given these atypical findings, A non-contrast T2-weighted MRCP was performed, demonstrating non-visualization of the gallbladder, a finding consistent with gallbladder agenesis (Fig. 2). A short blind-ending tubular structure, interpreted as a cystic duct remnant, was also observed (Fig. 3). The common bile duct (CBD) was dilated, measuring up to 10.4 mm (Fig. 4). No ectopic gallbladder or other biliary tract anomalies were detected. Furthermore, no communication between the presumed cystic duct remnant and the biliary tree could be demonstrated.

These findings established the diagnosis of GA. In the absence of complications, a conservative management strategy was adopted. The patient was counseled regarding the congenital nature of the condition and managed with dietary and symptomatic treatment. Dietary recommendations mirrored post-cholecystectomy advice, including smaller, more frequent meals and a low-fat diet, which was well tolerated. Smooth muscle relaxants were prescribed on an as-needed basis for pain control, without requirement for continuous therapy. No further pharmacological treatment was necessary given the mild symptom burden at presentation.

Follow-up for more than one year demonstrated sustained clinical improvement, with a marked reduction in the frequency and intensity of postprandial and right upper quadrant pain. A timeline table detailing the major clinical events of this case has been included to summarize the chronological sequence of presentation, diagnostic workup, and management (Table 2).

**Table 2:** Timeline of clinical, diagnostic, and therapeutic events

Time point	Event
August 2024	Biliary pain onset
February 2025	Primary consultation
February 2025	first follow up and First Us: non visualization of GB/retracted GB
March 2025	Second follow up and Second US: non visualization of GB, dilation of CBD
March 2025	Third follow up and MRCP: GB Agenesis with compensatory dilation of CBD
September 2025	Fourth Follow up: regression of symptoms
March 2026	Fifth Follow up: significant improvement in quality of life and symptoms

## Discussion

GA results from disruption of normal biliary embryogenesis and is a rare congenital anomaly characterized by complete absence of the gallbladder and often the cystic duct. During the fourth to seventh weeks of gestation, the gallbladder and cystic duct normally develop as a ventral outgrowth (the cystic bud) from the caudal portion of the foregut. Initially, the gallbladder primordium forms as a solid epithelial structure without a lumen. Subsequent vacuolization leads to recanalization and formation of the gallbladder lumen. Failure at any stage of this process—either absence of cystic bud formation or defective vacuolization and recanalization—may result in GA [7]. Its reported incidence in clinical and autopsy series ranges from approximately 10–65 cases per 100,000 individuals, with a female-to-male ratio of roughly 3:1 [8], although autopsy-based evaluations suggest higher estimates of around 90 cases per 100,000 [9, 10]. This rarity means GA may remain unrecognized in clinical practice, despite its relevance as a differential diagnosis in patients presenting with biliary symptoms.

The pathophysiology of symptoms in GA remains incompletely understood. Proposed mechanisms include sphincter of Oddi dysfunction, biliary dyskinesia, and increased intraductal pressure within the CBD [11, 12]. These functional disturbances explain why patients may present with classical biliary-type pain despite the absence of gallstones. Clinically, patients with GA can be categorized into three groups: asymptomatic individuals in whom the condition is discovered incidentally; symptomatic patients presenting with biliary colic or dyspeptic symptoms related to biliary dysfunction; and neonates in whom GA is associated with multiple congenital anomalies and often presents early in life [13].

Ultrasonography remains the first-line imaging modality for biliary pathology; however, in GA it is frequently misleading, often reporting a contracted, scleroatrophic, or shrunken gallbladder. This misinterpretation has historically resulted in unnecessary surgical exploration [14–16], during which the gallbladder is not identified, thereby increasing the risk of bile duct injury due to extensive dissection [17]. Consequently, diagnosis has often been established intraoperatively in reported cases, with MRCP subsequently performed for confirmation. Repeating ultrasonography may help clarify the diagnosis, preferably with a different operator; however, MRCP remains the diagnostic modality of choice [18, 19], as it allows accurate delineation of biliary anatomy and reliable exclusion of an ectopic gallbladder. In the present case, CBD dilation may represent a compensatory adaptation in the absence of a gallbladder reservoir, as the CBD can function as a bile storage pathway [20]. Mild age-related increase in CBD diameter has also been described in older patients without biliary obstruction [21]. Based on these findings, an obstructive etiology was considered unlikely. Similarly, a sphincteric dysfunction of Oddi’s was deemed improbable in the absence of biochemical cholestasis or radiological evidence of biliary obstruction, further establishing that the dilated CBD consists with non-obstructive adaptation, supporting preserved sphincter function.

A short cystic duct remnant measuring approximately 7 mm (Fig. 4) was identified, likely representing an incompletely developed cystic duct secondary to arrested embryological development of the gallbladder bud. This finding is consistent with observations from the literature, where laparoscopic exploration was abandoned early and only a short cystic remnant without identifiable ductal or arterial structures was noted [22].

**Fig. 5:**
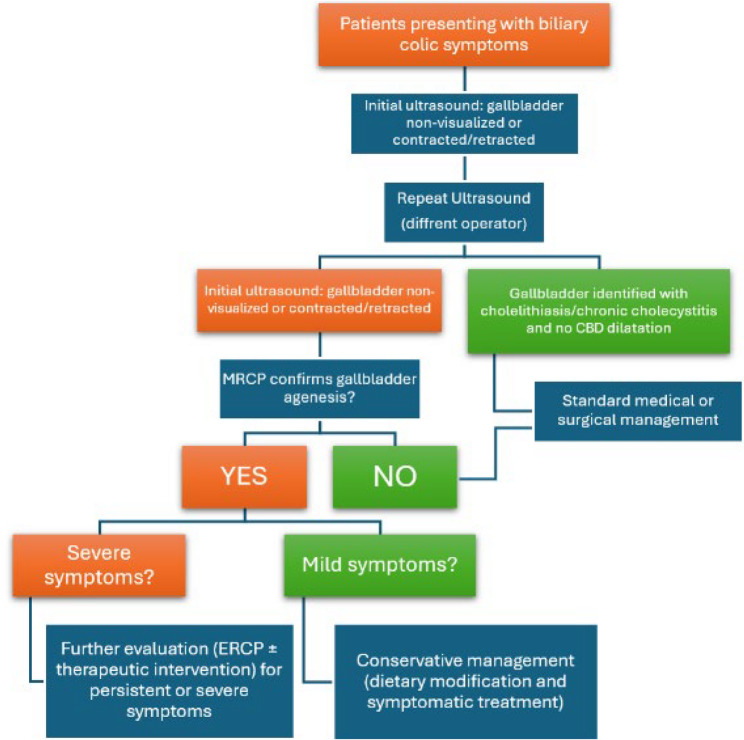
Suggested diagnostic and management algorithm for patients with suspected GA presenting with biliary symptoms

Atypical biliary imaging findings should prompt further radiological evaluation, even in patients presenting with otherwise routine biliary pain. In our case, ultrasound findings associated with CBD dilation led to MRCP, enabling preoperative diagnosis of GA and successful conservative management. In contrast, the case reported by Bahraini et al. [17] involved inconclusive preoperative imaging, including a non-diagnostic HIDA scan, followed by extensive intraoperative dissection in search of the gallbladder, resulting in CBD injury, conversion to open surgery, and Roux-en-Y hepaticojejunostomy reconstruction. Such major biliary injuries may carry significant short- and long-term morbidity. These observations support the role of MRCP prior to exploratory or therapeutic surgical intervention whenever uncertainty regarding biliary anatomy persists. In addition, a concise diagnostic and management algorithm has been included to illustrate the proposed stepwise approach when GA is suspected, aiming to reduce unnecessary surgical exploration and improve preoperative diagnostic accuracy (Fig. 5).

Recognition of GA before surgical intervention is essential. In most reported cases, surgeons do not convert to open surgery when the gallbladder is not identified intraoperatively; instead, the procedure is abandoned and MRCP is requested to confirm the diagnosis [9, 12, 14, 15]. Therefore, patients presenting with biliary symptoms and ultrasound findings suggestive of abnormal biliary anatomy such as a poorly visualized, or absent gallbladder, especially when associated with CBD dilation, should raise early suspicion and prompt preoperative MRCP, which is preferable to postoperative diagnosis or recognition after iatrogenic bile duct injury. Management GA is not standardized and remains without consensus due to its rarity. In symptomatic patients, a conservative approach is generally adopted and may include dietary modification and pharmacological treatment with smooth muscle relaxants or antispasmodics. In selected cases with persistent or severe biliary-type symptoms, endoscopic sphincterotomy has been described as a therapeutic option [3].

### Strengths

This case highlights the importance of considering GA in the differential diagnosis of biliary colic and emphasizes the role of preoperative MRCP in preventing unnecessary surgical exploration. A key strength lies in preoperative radiological confirmation, which allowed avoidance of potentially hazardous intraoperative dissection and reduced the risk of iatrogenic bile duct injury. Additionally, the case reinforces the limitations of ultrasonography and the diagnostic reliability of MRCP in suspected biliary anomalies.

### Limitations

However, this report is limited by its single-case design, restricting generalizability. Furthermore, the absence of intraoperative findings precludes direct surgical correlation of imaging results. As with most rare congenital anomalies, the literature remains largely based on case reports and small series, limiting definitive conclusions regarding optimal management strategies and long-term outcomes.

### Patient perspective

The patient initially believed she had gallbladder lithiasis. Following repeated imaging assessments and review, the diagnosis of GA was established. She expressed concern regarding the potential severity of her condition; however, these concerns were subsequently alleviated after clinical evaluation and explanation of the diagnosis. With adherence to dietary modifications and conservative management, she reported early and sustained improvement in symptoms shortly after initiation of treatment.

## Conclusion

GA, although rare, should be considered in the differential diagnosis of patients presenting with biliary-type pain when the gallbladder is not visualized on ultrasonography. In such cases, cross-sectional imaging, particularly MRCP, plays a central role in confirming the diagnosis and accurately delineating biliary anatomy, thereby reducing diagnostic uncertainty. Early recognition of this congenital anomaly is essential, as failure to identify it preoperatively may lead to unnecessary surgical exploration and potentially avoidable iatrogenic injury. This case supports non-operative management as the primary approach in uncomplicated patients, with symptom-directed therapy and patient education associated with favorable outcomes. Greater awareness among surgeons, emergency physicians, and radiologists is therefore warranted to promote appropriate imaging pathways and avoid unnecessary intervention.

## Data Availability

No datasets were generated or analysed during the current study.

## Abbreviations

GA: Gallbladder agenesis
HIDA: Hepatobiliary Iminodiacetic Acid scan
MRCP: Magnetic Resonance Cholangiopancreatography
ERCP: Endoscopic Retrograde Cholangiopancreatography
CBD: Common bile duct
GGT: Gamma-glutamyl transferase
